# Association between white matter impairment and cognitive dysfunction in patients with ischemic Moyamoya disease

**DOI:** 10.1186/s12883-020-01876-0

**Published:** 2020-08-15

**Authors:** Ziqi Liu, Shihao He, Zongsheng Xu, Ran Duan, Li Yuan, Chu Xiao, Zhe Yi, Rong Wang

**Affiliations:** 1 Department of Neurosurgery, Beijing Tiantan Hospital, Capital Medical University, 119 South Fourth Ring West Road, Fengtai District, Beijing, 100070 China; 2 Center of Stroke, Beijing Institute for Brain Disorders, Beijing, 10069 China; 3 Department of Neurosurgery, Peking University International Hospital, Beijing, 102206 China; 4 State Key Laboratory of Cognitive Neuroscience and Learning & IDG/Mc Govern Institute for Brain Research, Beijing Normal University, Beijing, 100875 China; 5 Jishuitan Hospital, Fourth Clinical College of Peking University, Beijing, 100096 China

**Keywords:** Moyamoya disease, Cognitive dysfunction, Diffusion tensor imaging, Tract-based spatial statistics, Ischemic cerebrovascular disease

## Abstract

**Background:**

Ischemic Moyamoya disease is one of the important causes of stroke, which leads to severe impairment in cognitive functions. This cognitive impairment occurs prior to stroke. However, the cognitive functions that are impaired and the mechanisms of these impairments have not been determined.

**Methods:**

We analyzed 12 patients with Moyamoya disease and 12 controls. All participants underwent cognitive tests and magnetic resonance imaging (MRI) scans. The diffusion tensor imaging (DTI) data was processed using Tract-Based Spatial Statistics (TBSS). Significantly different white matter areas were correlated with different cognitive functions.

**Results:**

There were significant differences in intelligence and subtraction between the patients and controls (*p* < 0.05). The parameters of DTI such as fractional anisotropy (FA), mean diffusivity (MD), axial diffusivity (AD), and radial diffusivity (RD) have different changes in anterior thalamic radiation, inferior fronto-occipital fasciculus (IFO), superior longitudinal fasciculus (SLF), uncinate fasciculus (UF), inferior longitudinal fasciculus, forceps minor, and other regions between the two groups.

**Conclusion:**

Left UF and IFO may be the key brain regions affecting arithmetic function, while bilateral IFO has an effect on intelligence. RD and AD may be better indicators for early prediction of chronic white matter damage than FA, while MD tends to have a comprehensive indirect change. There is cognitive impairment in ischemic MMD, which is closely related to white matter impairment.

**Trial registration:**

Clinical Trial Registration, Unique identifier: ChiCTR1900023610. Registered 4 June 2019 – Prospective study registered.

## Background

Moyamoya disease (MMD) is a congenital cerebrovascular malformation that leads to progressive stenosis of the main arteries of the brain and compensatory proliferation of small puffy vessels in the base of brain [1], which eventually leads to serious cerebrovascular accidents, particularly stroke. Previously, the risk of dementia after stroke was reported to be as high as 30% for all kinds of diseases [2], while the risk of dementia before stroke was approximately 9–14% [3]. In a small sample study, about two-thirds of patients with MMD have cognitive impairment [4], however, not all patients with cognitive dysfunction suffer from stroke. MMD has a significant effect on the cognitive abilities of adults without stroke, and in about a quarter of patients [5], the degree of cognitive impairment may affect daily function. It is concerning that MMD patients without stroke have a high rate of dementia. The impairment of cognitive function in MMD has been widely studied. Previous studies have found that MMD patients have significantly lower executive function, attention, and short-term memory than controls [5–7], but the difference in the structure of the brain between patients and controls is not known. Neuroimaging is a uniquely noninvasive way to study brain structure. Therefore, we used neuroimaging to find a link between different parts of the brain and cognitive functions.

Neuroimaging, especially diffusion tensor imaging (DTI), is important for studying brain structure and function. It has been reported that DTI is highly sensitive to changes in the microstructure of white matter diffusion characteristics, which is mainly used for nervous system disease with white matter damage but rarely in MMD. Previously, Kazumata et al. [8] found that the mean fractional anisotropy (FA) of white matter tracts in the lateral prefrontal lobe, cingulate region, and inferior parietal region was significantly correlated with processing speed, executive function, and working memory. However, the number of such studies in MMD is limited. We aimed to study white matter fiber bundle damage in MMD patients without stroke by DTI to investigate whether the site of damage in white matter fiber bundles is associated with certain cognitive impairment and thus, determine the cause of cognitive impairment in patients without stroke.

## Methods

### Patients

This prospective study was approved by the research ethics committee of Beijing Tiantan Hospital affiliated to Capital Medical University (KYSQ2019–058-01). Written informed consent was obtained from all participants. The study included 12 patients with MMD (5 men, 7 women; mean age 42.83 ± 8.80 years old; mean education: 9.83 ± 4.09 years) from the Neurosurgery Department of Beijing Tiantan Hospital which is affiliated to the Capital Medical University and Peking University International Hospital between June 2019 and December 2019. Moreover, the control group included 12 volunteers (7 men, 5 women; mean age 39.33 ± 10.82 years old, mean education: 12.42 ± 3.42 years). There was no significant difference in sex composition, age, education level, and risk factors between the two groups (*P* > 0.05). Details of Suzuki Stage could be found in Tables 1 and 2.

**Table 1:** Basic information of patients and controls

	Patients (*n* = 12)	Controls (*n* = 12)	Statistics
Variables	Mean ± SD	Mean ± SD	*P* values
Sex (M:F)	05:07	07:05	0.414
Age (years)	42.83 ± 8.80	39.33 ± 10.82	0.394
Education	9.83 ± 4.09	12.42 ± 3.42	0.107
Medical history, n (%)			
Hypertension	2 (16.7)	2 (16.7)	1
Dyslipidemia	3 (25.0)	1 (8.3)	0.273
Smoking history	3 (25.0)	2 (16.7)	0.615
Alcohol taking	1 (8.3)	2 (16.7)	0.537
Suzuki Stage			
Left			
1	2 (16.7)		
2	2 (16.7)		
3	5 (41.7)		
4	2 (16.7)		
5	1 (8.3)		
6	0		
Right			
1	1 (8.3)		
2	1 (8.3)		
3	8 (66.7)		
4	1 (8.3)		
5	1 (8.3)		
6	0		

Values are numbers of cases (%) unless otherwise indicated. Mean values are presented with SDs

*Abbreviations: F* female, *M* male, *SD* standard deviation

**Table 2:** Patients characteristics

Sex	Education (year)	Clinical Presentation	Suzuki Grade		Medical History	Vessel Stenosis or Occlusion Pattern	
			Left	Right		Left	Right
F	15	TIA	3	4	None	ACA* MCA*	ICAO* MCA*
F	9	Asymptomatic	2	3	None	MCA	MCA
M	12	Asymptomatic	1	3	Hypertension	ICAS	ICAS MCA
F	4	Asymptomatic	3	1	None	ACA* MCA*	ICAS
F	8	TIA	3	3	None	ACA*	MCA*
F	3	TIA	1	3	Dyslipidemia	ICAS	ACA MCA
M	15	TIA	3	3	None	ACA MCA	ACA MCA
F	9	TIA	5	3	None	ICAO*	ICAO*
M	9	TIA	3	3	Dyslipidemia	ICAS ACA MCA	MCA*
					Hypertension		
F	16	TIA	4	5	None	ICAO* ACA MCA	ICAO* ACA MCA
M	9	Asymptomatic	4	3	None	ICAO*	ICAS ACA MCA
M	9	Asymptomatic	2	2	Dyslipidemia	MCA	ICAS

*Abbreviations: F* female, *M* male, *TIA* transient ischemic attack, *ACA* anterior cerebral artery, *MCA* middle cerebral artery, *ICAS* internal carotid artery stenosis, *ICAO* internal carotid artery occlusion;

*The asterisk stands for occluded vessels. (for example: ACA* means anterior cerebral artery occlusion)

The black word stands for narrow vessels. (for example: MCA means middle cerebral artery stenosis)

The inclusion criteria of patients were as follows:(1) All patients should meet the Guidelines for Diagnosis and Treatment of Moyamoya Disease (Spontaneous Occlusion of the Circle of Willis), the research committee on the pathology and treatment of spontaneous occlusion of the circle of willis; health labour sciences research grant for research on measures for intractable diseases [9]; (2) In the MMD patients group, there was no previous ischemic or hemorrhagic attack, and in the case of intracranial lacunar cerebral infarction, the lesion area should be less than 1.5 cm; (3) righthand dominance; (4) being free of dementia, or depression; and (5) no major psychiatric disease or other medical conditions.

The exclusion criteria of patients were as follows: (1) Acute stage of cerebral infarction and other neuropsychiatric diseases, severe systemic diseases, and severe systemic diseases (e.g., AD, Parkinson’s disease); (2) any contraindications for MR scans (e.g., metal implants); (3) Manifestation of any medications that could affect cognitive abilities; (4) fatigue or hunger; or (5) an inability to complete the tasks independently.

By means of social recruitment, we released recruitment advertisements for the control group. A total of 20 people was included in the control group. Through asking medical history, we recorded clinical variables such as age, sex and past medical history, and conducted cognitive test after MRI examination. 8 people were excluded through exclusion criteria because of brain cysts (1), incomplete cognitive test (3), large head movement or low signal-to-noise ratio (SNR) during MRI scanning (4). Twelve people were finally included in the control group and matched with the MMD group.

Inclusion criteria for the control group were as follows: no clinical evidence of psychiatric or neurological disease, no brain damage on routine MRI, and no history of drugs usage that could affect cognitive function.

### MRI acquisition

MRI data were obtained using a 3.0-Tesla MR system (Verio A Tim + Dot System, Siemens, Germany). Volumetric T1 (three-dimension, 3D) gradient echo was acquired in the sagittal plane with a thickness of 1 mm (flip angle = 8, time of repetition (TR) = 2300 ms, time of echo (TE) = 3.25 ms, matrix =256 × 256, FOV = 250 × 250 mm). DTI following echo planar imaging (EPI) was acquired in 30 directions (flip angle = 180°, voxel size = 1.8 × 1.8 × 4.0 mm^3^, TR =3600 ms, TE = 95 ms, matrix =128 × 128, FOV = 230 × 230 mm, 25 cuts with 4 mm thickness, b-value = 1000).

### Tract-based spatial statistics analysis

DTI preprocessing used PANDA [10] pipeline which was conducted through FMRIB Software Library (FSL 5.0.9, University of Oxford, UK, http://www.fmrib.ox.ac.uk/fsl). Firstly, in the raw DTI images, using the FSL Eddy Correction Tool [11], eddy current distortions and motion artifacts were corrected. Then, the corrected DTI images were stripped to remove non-brain tissues like the skull and muscle by the FSL Brain Extraction Tool (BET) [12]. Secondly, each individual images, including FA, mean diffusivity (MD), and three eigenvalues λ_1_, λ_2_ and λ_3_, were calculated using the FSL diffusion tensor analysis toolkit (FDT) [13]. Axial diffusivity (AD) was generally accepted as the largest eigenvalue (λ_1_), while radial diffusivity (RD) was defined as the mean of the two smaller eigenvalues (λ_2_ and λ_3_). Then MD was calculated as the mean of the three eigenvalues (λ_1_, λ_2_, and λ_3_). Next, we analyzed the FA images of all the patients and controls in the Tract-Based Spatial Statistics (TBSS) analysis [14] within FSL following the standard pipeline (www.fsl.fmrib.ox.ac.uk/fsl/fslwiki/TBSS). All FA images were nonlinear-registered to Montreal Neurological Institute 152 (MNI 152) space through the FSL registration tool FNIRT, and the mean FA images and skeleton (FA threshold 0.2) were created then. Finally, all participants’ FA images were projected onto this skeleton to create normalized skeletonized FA images. Then, similarly, through the nonlinear transformation of FA images, AD, MD, and RD images were all registered to the Montreal Neurological Institute (MNI) standard space and individual skeletonized images were generated for next analysis.

### Cognition acquisition

All cognitive assessment programs were tested using the Online Psychological Experimental System. Choice reaction time (RT) was used as the baseline condition and the index of movement ability. The objective of using the basic RT task was to determine manual response effect and main processing speed. The choice RT task was adapted from the simple RT task from Butterworth’s Dyscalculia Screener [15]. In all 30 trials of this task, a white fixation cross and a white dot were presented on a black screen. The former was presented in the center of the screen, and the latter was presented on the left or right side of the fixation cross (It was present on the left side in half the cases). Participants should press “Q” or “P” with their left or right fingers if the dot appeared on the left or right side of the fixation cross, respectively. The interstimulus interval was randomized between 1500 and 3000 ms. Nonverbal matrix reasoning was used to assess general intelligence and reasoning ability. The task was adapted from the abstract reasoning ability part of Raven’s Progressive Matrices (Raven, 2000). The mental rotation was used to evaluate the visual-spatial ability [16]. Verbal working memory was used to measure working memory capacity [17]. A multistep, multilocation search task was used to evaluate executive function [18]. Simple and complex subtraction problems were used to assess simple calculation ability and magnitude representation [19]. Word-memory ability and visual short-term memory were measured with the short-term memory (STM) span for Chinese words and phrases and the picture STM test, respectively [20]. The Edinburgh Handedness Inventory was used to investigate left and right-handedness [21]. The AD8 questionnaire was used to determine the degree of cognitive decline in daily life [22]. Additional details on the cognitive assessment questions can be found elsewhere [23]. The participants were tested using computer workstations by neuropsychologists who were blinded to the clinical data. The interval between neuropsychological testing and MRI examination was < 5 days.

### Statistical analysis

Voxel-wise statistics across participants were put into effect for each voxel of FA images. We used 5000 permutations and Threshold-Free Cluster Enhancement (TFCE) to correct multiple comparisons. Considering results of the voxel-wise analyses, we reported the significant clusters ≥15 voxels, labeled them according to the Johns Hopkins University JHU-ICBM-tracts atlas. Then we binarized the TFCE corrected statistical maps into masks (uncorrected *p* < 0.05). Finally, Pearson correlation analysis was performed between the significant cognitive scores and clusters of white matter fibers for patients and healthy controls, (p < 0.05) [24] in SPSS 19.0 (IBM Corp. Released 2010. IBM SPSS Statistics for Windows, Version 19.0. Armonk, NY: IBM Corp.). Similarly, we repeated the same analyses for the AD, MD, and RD values, but we binarized the TFCE corrected statistical maps into masks with corrected *p* < 0.05.

## Results

### Cognition result

Cognitive tests were performed on 12 MMD patients and 12 controls, and it was found that the function of Raven’s Standard Progressive Matrices (SPM), Mental rotation (ROT), verbal working memory 2(VWM2), Simple subtraction (SUB), Complex subtraction (COMSUB), and word-memory (WORDM) in the patient group were all significantly lower than that in the control group (p < 0.05). Moreover, there were very significant differences in RAVEN, VWM2, COMSUB, and WORDM functions between the patients and the control group (*p* < 0.01). Moreover, it is noteworthy that, although differences in VWM1 (*p* = 0.053) and ANXIETY (*p* = 0.062) were insignificant, there was a decline in the scores in the patient group. More cognitive details could be found in Table 3.

**Table 3:** Summary of neuropsychologic assessments in each group

Variables	Patients (*n* = 12)	Controls (*n* = 12)	Statistics
	Mean ± SD	Mean ± SD	*P* values
AD-8	1.08 ± 1.311	0.42 ± 0.67	0.131
CRT_RT	999.25 ± 809.84	483.42 ± 249.89	0.055
CRT_ACC	93.00 ± 17.29	99 ± 1.48	0.256
SPM	13.92 ± 4.91	25.17 ± 8.56	0.001**
ROT	12.08 ± 5.65	18.33 ± 7.40	0.030*
VWM1	7.33 ± 1.88	8.67 ± 1.23	0.053
VWM2	5.42 ± 1.51	7.83 ± 1.64	0.001**
SUB	29.92 ± 14.50	43.83 ± 9.18	0.011*
COMSUB	13.50 ± 6.65	24.33 ± 6.07	0.000**
WORDM	55.67 ± 14.27	69.67 ± 9.22	0.009**
PICTM	68.33 ± 17.97	76.33 ± 4.33	0.148
EXCUT1	0.58 ± 2.39	−0.83 ± 2.89	0.204
EXCUT2	−1.67 ± 4.05	−3.00 ± 2.26	0.333
ANXIETY	1.92 ± 2.28	0.50 ± 0.80	0.062
DEPRESS	0.50 ± 0.91	0.17 ± 0.39	0.259

*Abbreviation: SD* standard deviation, *CRT_RT/ACC* Choice reaction time_ reaction time/ accuracy, *SPM* Raven’s Standard Progressive Matrices, *ROT* Mental rotation, *VWM* verbal working memory, digit span, 1, Recite in order, 2, Recite in reverse order, *SUB* Simple subtraction, *COMSUB* Complex subtraction, *WORDM* word-memory, *PICTM* picture-memory, *EXCUT* Executive function,1, same direction, 2, Opposite direction, *ANXIETY*, Hamilton Anxiety Scale, *DEPRESS* Hamilton Depression Scale *p < 0.05, **p<0.01

### Microstructural changes in white matter fibers

**Fig. 1:**
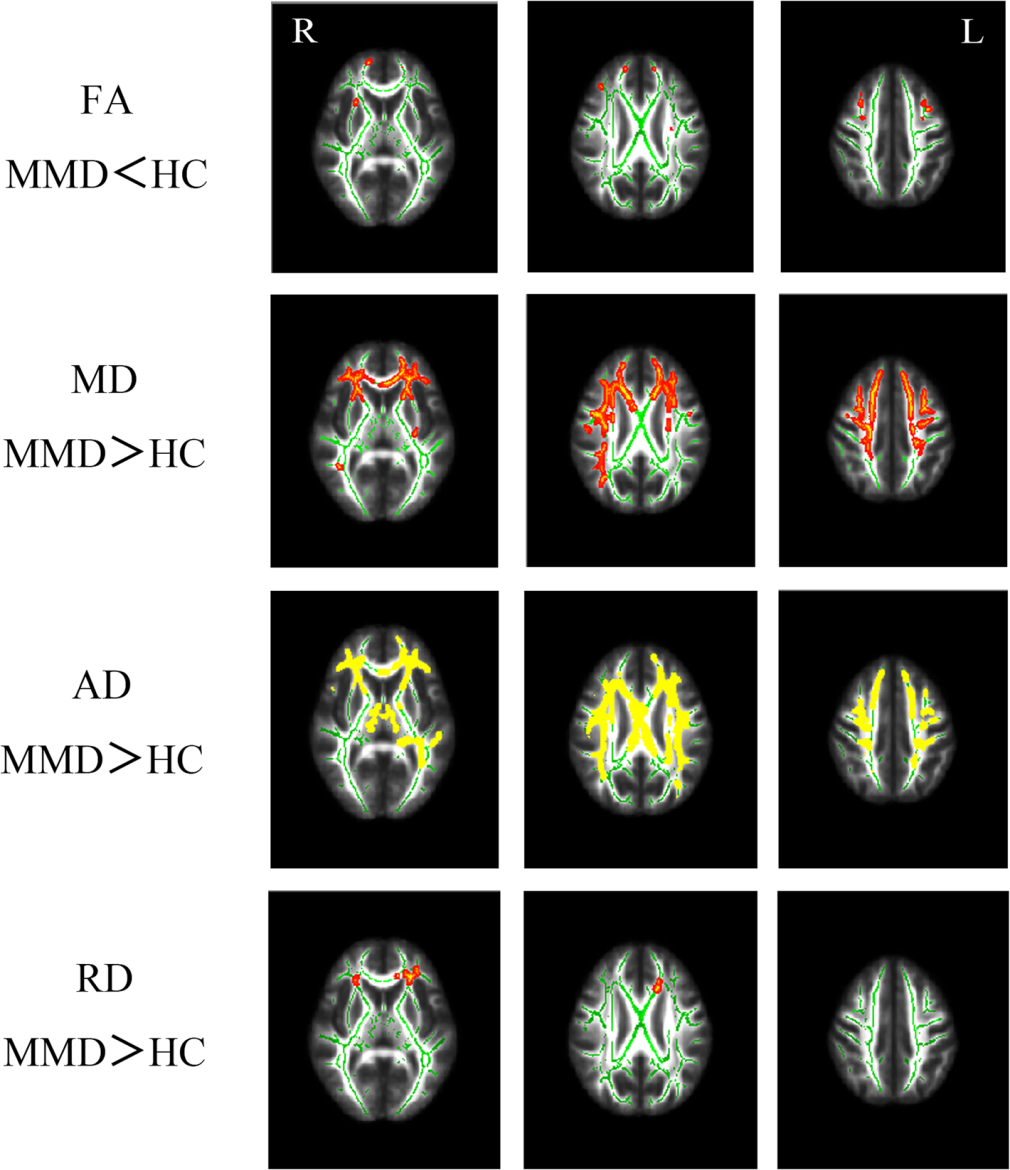
Differences in FA, MD, RD, and AD values in different regions of the white matter between the patient group and control group after 5000 permutations and TFCE corrected *p*<0.05 (MD, AD, RD) or TFCE uncorrected *p*<0.05 (FA)

Compared with the control group, we found changes in FA, MD, AD, and RD in the patient group. With 5000 permutations and TFCE, we found that the FA value differences between patient group and control group were found in the forceps minor, right anterior thalamic radiation (ATR), and right frontal occipital fasciculus (MMD<healthy controls (HC), TFCE uncorrected *p* < 0.05). The differences in MD, AD, and RD values were more extensive than the differences in FA values. MD and AD values of the patient group in forceps minor, bilateral superior longitudinal fasciculus (SLF), bilateral ATR, bilateral inferior frontal-occipital fasciculus (IFO), and left uncinate fasciculus (UF) were higher than those in the control group (TFCE corrected *p* < 0.05); RD values of the patient group were higher in the forceps minor, left IFO, left ATR, and left UF than those in the control group (TFCE corrected p < 0.05). The details are shown in Fig. 1 and Table 4.

**Table 4:** Significantly different white matter regions

Index		Number of voxels	Peak coordinate (MNI)			Label
			X	Y	Z	
FA	MMD < HC	39	−17	50	12	forceps minor
		23	38	33	27	ATR R
		23	15	57	5	IFO R
		22	−9	−17	67	SLF L
		19	28	17	4	SLF R
		18	27	21	0	UF R
MD	MMD > HC	^a^				forceps minor
						SLF R
						ATR L
						ATR R
						IFO L
						IFO R
						UF L
AD	MMD > HC	^a^				forceps minor
						ATR L
						ATR R
						SLF L
						SLF R
						IFO L
						IFO R
						ILF L
						UF L
RD	MMD > HC	^a^				IFO L
						ATR L
						forceps minor
						UF L

Table 4: DTI index and significant different cluster in JHU-ICBM-tracts atlas and peak coordinate in MNI. *Abbreviation: ATR* anterior thalamic radiation, *IFO* inferior frontal-occipital fasciculus, *SLF* superior longitudinal fasciculus, *UF* uncinate fasciculus, *ILF* inferior longitudinal fasciculus, *L* left, *R* right. ^a^The voxels of the cluster which is combined with several regions are too large to locate the peak coordinate

### Correlation of DTI index and cognition

We analyzed the correlation between DTI indicators, including FA, MD, AD, and RD values, and cognitive function with significant differences between the patient and control groups.

In the patient group, there was a negative correlation between Raven reasoning test and MD in bilateral IFO (left *r* = − 0.645, *p* = 0.023; right *r* = − 0.73, *p* = 0.007), right SLF (*r* = − 0.585, *p* = 0.046), and left UF (*r* = − 0.576, *p* = 0.050). Moreover, Raven reasoning test and AD of right SLF (*r* = − 0.673, *p* = 0.016) were negatively correlated. Simple subtraction and MD of left ATR (*r* = − 0.642, *p* = 0.024), left IFO (*r* = − 0.686, *p* = 0.014), and left UF (*r* = − 0.669, *p* = 0.017) were negatively correlated. Simple subtraction and RD of left ATR (*r* = − 0.656, *p* = 0.021), left IFO (*r* = − 0.748, *p* = 0.005), and left UF (*r* = − 0.622, *p* = 0.031) were negatively correlated as well. The complex subtraction and MD of left IFO (*r* = − 0.651, *p* = 0.022) and left UF (*r* = − 0.623, *p* = 0.031) and RD of left IFO (*r* = − 0.697, *p* = 0.012) were negatively correlated.

**Fig. 2:**
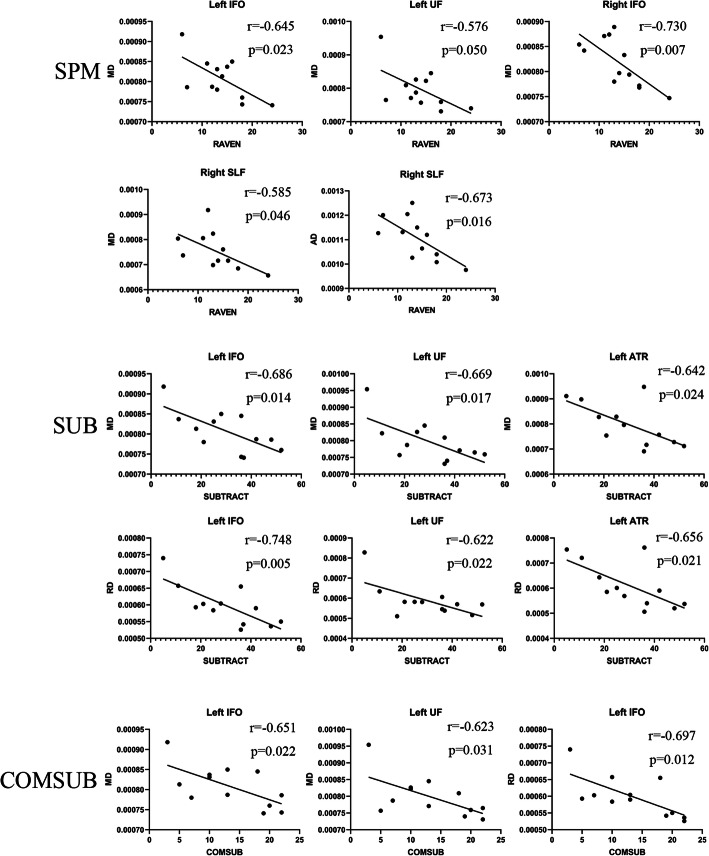
Correlation of cognition and WM regions in MMD group

**Fig. 3:**
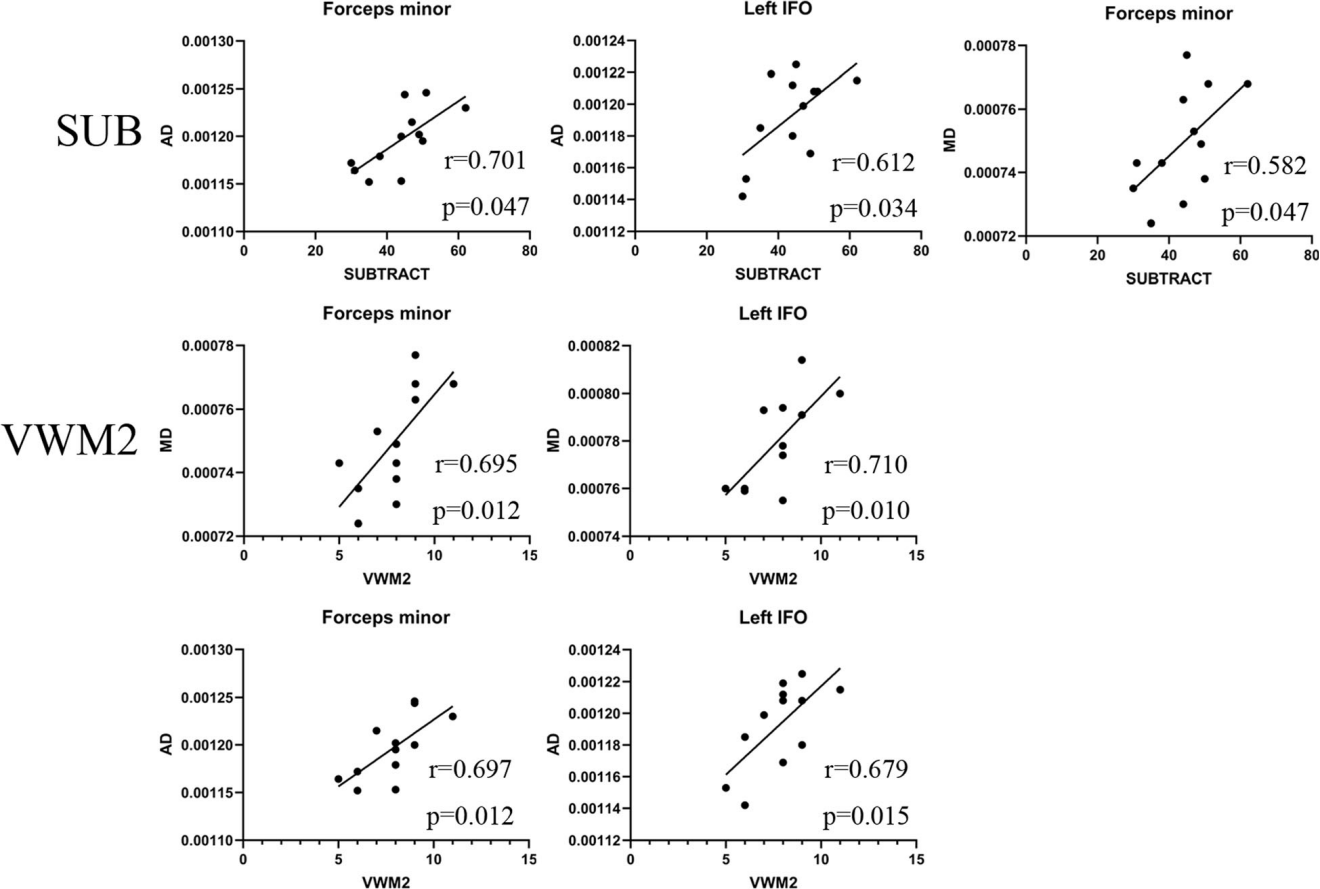
Correlation of cognition and WM regions in control group

In the control group, we found that the simple subtraction and AD of left IFO (*r* = 0.612, *p* = 0.034) and forceps minor (*r* = 0.701, *p* = 0.011) was positively correlated; additionally, simple subtraction and MD of forceps minor (*r* = 0.582, *p* = 0.047) was positively correlated. Verbal working memory 2 and MD value of left IFO (*r* = 0.710, *p* = 0.010) and forceps minor (*r* = 0.695, *p* = 0.012) was positively correlated. Moreover, verbal working memory 2 and AD value of left IFO (*r* = 0.679, *p* = 0.015) and forceps minor (*r* = 0.697, *p* = 0.012) was positively correlated. Other brain regions were not found to be correlated with each index of DTI. The result is shown in Fig. 2 and Fig. 3.

## Discussion

Diffusion tensor imaging (DTI) has been proven to be an effective method for detecting white matter integrity and exploring the relationship between brain microstructure and cognitive function [25–28]. This is one of the few studies on white matter and cognitive function in asymptomatic ischemic MMD patients. Moreover, our analysis method and the patient selection criteria are more reasonable than that of previous studies, and we found asymptomatic ischemic MMD white matter lesions and cognitive changes. We have performed and explained each of the correlation analysis.

In the cognitive results, we found that the main cognitive impairments in the MMD group were mainly in the areas of logical reasoning, short-term memory, executive function, and calculation, which was similar to that reported in previous studies; however, our analysis was detailed. Previous studies have found that MMD patients have significantly lower executive function, attention, and short-term memory than normal control groups. Long-term ischemia in MMD patients leads to cognitive impairment that includes selective impairment of these cognitive functions. MMD mostly causes narrowing or occlusion of the internal carotid artery, middle cerebral artery, and anterior cerebral artery, and the regions supported by these vessels are mostly located in the anterior half of the brain, including the frontal lobe, temporal lobe, and parietal lobe. The main aim of this study was to analyze whether there was substantial damage to the white matter in these areas.

While analyzing the differences in white matter fiber microstructure between the MMD group and the control group, we observed changes in FA, MD, AD, RD, and other indicators. In general, compared with the control group, the MMD group showed a decrease in FA and an increase in MD, AD, and RD values, but brain area with reduced FA was relatively small (TFCE uncorrected *p* < 0.05), while the MD, AD, and RD showed differences over a wide range of brain area in the patient group (TFCE corrected p < 0.05). This is a very interesting phenomenon. According to previous studies, RD and AD are thought to be more sensitive measures of neurodegeneration changes than FA [29]. AD value is highly sensitive to the maturation of white matter and the increase in water components, while RD value is closely related to demyelination changes and myelin sheath diameter. MD is a reflection of cellularity, edema, and necrosis [30–32]. We suspect that the local decrease in FA values observed in MMD patients and the increase in MD, RD, and AD values over a wide area are due to a change caused by the increase of cellulose water composition caused by ischemia and the damage of myelin sheath. First, the pathological process of MMD is considered to be a kind of ischemic change, and long-term brain main blood supply artery ischemia is bound to lead to overall brain ischemia and hypoxia. Although the damage at the cellular level is unknown, but this kind of chronic ischemia may cause structural changes in the cells, and this is consistent with an increase in the MD, AD and RD values. Therefore, we have reason to suspect that MMD ischemic injury can damage the myelin sheath and affect the transmission function of white matter fiber tracts, and thus, affect its areas of cognitive function. In the early days of the change, mainly AD and RD values change, and the increase in MD may be due to indirect reasons, while the FA value does not change as most of the cognitive functions are reserved. In addition, it is worth mentioning that both FA and MD change with age [33–35], therefore, age factors were considered in the inclusion of patients and controls to exclude the influence of age.

Previous studies by Kazumata et al. [36] found that the changes in FA, MD, and RD parameters in MMD patients were relatively extensive, while there was almost no change in AD parameters. This is not entirely consistent with our results. Although all patients were selected as asymptomatic ischemic MMD patients, there was a significant difference in disease risk between the enrolled patients and the control group, and the DTI analysis adopted the method of self-selecting ROI in the SPM8. However, there were no significant differences in age, sex, education level, and risk between the patient group and the control group, excluding diseases such as diabetes that may cause damage to white matter. Furthermore, we used TBSS to analyze DTI data, which is more technically advanced than SPM8. Although there are several differences, many of the results are similar. For example, Kazumata et al. found that the DTI index changes in the knee and bilateral longitudinal bundle of the corpus callosum, which coincides with our results.

The correlation analysis of cognition and DTI parameters showed that the cognitive function reduction of MMD patients mainly focuses on Raven’s reasoning test, subtraction, and complex subtraction. Therefore, we mainly discussed the arithmetic ability and logical reasoning ability of patients.

Math calculation is a complex skill and not a simple process [37–39]. It requires many cognitive processes, including attention, working memory, and processing speed, in addition to specific mathematical skills. Previous studies have reported that math calculation was associated with atypical brain function as well as atypical brain structure and connectivity [40]. The study of Navas-Sanchez et al. [41] on 13 mathematically gifted children and 23 controls found that in the mathematically gifted children, the FA value increased in the bilateral SLF, IFO, ATR, and left UF. Li et al. [42] found that the FA value reduction of left ILF and bilateral IFO was negatively correlated with the decline of calculation ability in the study of mathematical subtraction ability of 47 children. In the study of 30 adults with dyscalculia and 17 controls, Kucian et al. [43] found that the FA value of bilateral SLF in dyscalculia adults was reduced, and this correlated with mathematical ability. In our study, the FA value of these regions in MMD patients decreased, but was not correlated with mathematical subtraction. On the contrary, in the left ATR, IFO, and UF, there was a significant negative correlation between RD and MD values and mathematical subtraction ability. Additionally, this indicates that RD value may be a more sensitive indicator than FA value. In the previous meta-analysis [13], it was found that the higher the MD value in the UF region, the worse the attention, processing speed, and working memory. Although this does not explicitly point to mathematical power, it will undoubtedly have an impact on math calculation. Many studies [44–47] have reported a correlation between the left hemisphere, but not the right hemisphere, and computational power, which we speculate may be related to right-handedness and the dominant hemisphere.

The Raven’s test is a purely nonverbal intelligence test. In previous studies for 16 adolescents [48], there was a significant positive correlation between FA value of the right suboccipital tract and IQ. Although we did not observe the intelligence related to the FA values in these areas, a decrease in FA, MD, and AD values was observed in the left ILF and right IFO. Furthermore, in the correlation analysis, a negative correlation between MD values of bilateral IFO, left UF, and right SLF and raven score was observed.

## Limitations

Our research has some limitations. First, the sample size of this study is not enough. Due to infarction and hemorrhage in MMD, inclusion and exclusion criteria limit the number of patients. Although there was no statistical difference between patients and the controls, it is not so well matched with controls (control group has more males, younger patients and more schooling). Larger numbers of matched controls were needed for better homogeneity. Second, for some cognitive test with several significant differences, such as the rotation, we did not find associated brain regions to explain the differences. Third, although we included patients with bilateral MMD, the occlusive degree and location of bilateral vessels in these patients was not uniform, and the small cohort limited further stratification. Moreover, previous studies have proved that DKI parameters, such as MK, are more sensitive than DTI parameters. Therefore, we considered adding DKI scanning to improve our project. Finally, we would like to have a comparison before and after surgery to see if there is a possibility of improvement in these different brain regions. These studies are already under way.

## Conclusion

Ischemic MMD has a unique effect on the cognitive function of the brain, especially in arithmetic and intelligence. Left UF and IFO may be the key brain regions affecting arithmetic function, while bilateral IFO has an effect on intelligence. Long-term chronic ischemia has damage to the white matter of both sides of the brain, but the left hemisphere is more serious than the right hemisphere. RD and AD may be better indicators for early prediction of chronic white matter damage than FA, while MD tends to be a represent a comprehensive indirect change. All the above evidences indicate that there is cognitive impairment in ischemic MMD, which is closely related to white matter impairment.

## Data Availability

The datasets used and analysed during the current study are available from the corresponding author on reasonable request.

## Abbreviations

FA: fractional anisotropy
AD: axial diffusivity
RD: radial diffusivity
MD: mean diffusivity
WM: White matter
MMD: Moyamoya Disease
DTI: Diffusion tensor imaging
TBSS: Tract-Based Spatial Statistics
ATR: anterior thalamic radiation
IFO: inferior fronto-occipital fasciculus
SLF: superior longitudinal fasciculus
UF: uncinate fasciculus
ILF: inferior longitudinal fasciculus
